# Ovarian Hyperstimulation Syndrome as an Etiology of Obstructive Uropathy

**DOI:** 10.1155/2013/653704

**Published:** 2013-06-26

**Authors:** Creticus P. Marak, Amit Chopra, Narendrakumar Alappan, Ana M. Ponea, Achuta K. Guddati

**Affiliations:** ^1^Division of Pulmonary and Critical Care Medicine, Montefiore Hospital, Albert Einstein College of Medicine, Yeshiva University, New York, NY 10467, USA; ^2^Department of Internal Medicine, Massachusetts General Hospital, Harvard Medical School, Harvard University, Boston, MA 02114, USA

## Abstract

Ovarian hyperstimulation syndrome (OHSS) is an iatrogenic complication of controlled ovarian hyperstimulation (COH) protocols performed in women undergoing assisted reproductive technologies. Overstimulation of the ovaries results in the overproduction of vasoactive cytokines and mediators by the ovaries, thereby causing a generalized capillary leak and acute shift of protein-rich fluid from the intravascular compartment into the third space. This may lead to the development of ascites, pleural effusions, pericardial effusion, anasarca, intravascular volume depletion, hemoconcentration, oliguria, hypoalbuminemia and hypoproteinemia, electrolyte imbalances, acute renal failure, abdominal compartment syndrome, thromboembolic events, and adult respiratory distress syndrome. The only effective treatment available is prevention of the syndrome from developing by individualizing the stimulation protocol, especially in high-risk patients. Once the syndrome develops, the management is mainly supportive. Oliguria and some degree of acute renal failure commonly develop in patients with moderate to severe OHSS and are usually due to prerenal causes. Acute renal failure (ARF) secondary to obstructive uropathy is rare. Here we report a case of severe, life-threatening OHSS resulting in ARF secondary to obstructive uropathy.

## 1. Introduction

OHSS is a potentially life-threatening complication of ovulation induction. The syndrome is characterized by the presence of enlarged ovaries (due to enlarged follicles) and an acute shift of protein-rich fluid out of the vascular compartment. This syndrome occurs almost exclusively during assisted reproductive technology (ART) cycles, although cases occurring after ovarian stimulation using clomiphene citrate, spontaneous pregnancy (especially molar pregnancy and multiple pregnancies), and mutations involving FSH receptors have been described [1, 2]. Various mediators have been implicated in the pathogenesis of OHSS; these include interleukins (IL1, IL2, IL6, IL8, endothelin 1, TNF-alpha), prostaglandins and renin angiotensin aldosterone system, and vascular endothelial growth factor (VEGF) [3].

## 2. Case Report

**Figure 1:**
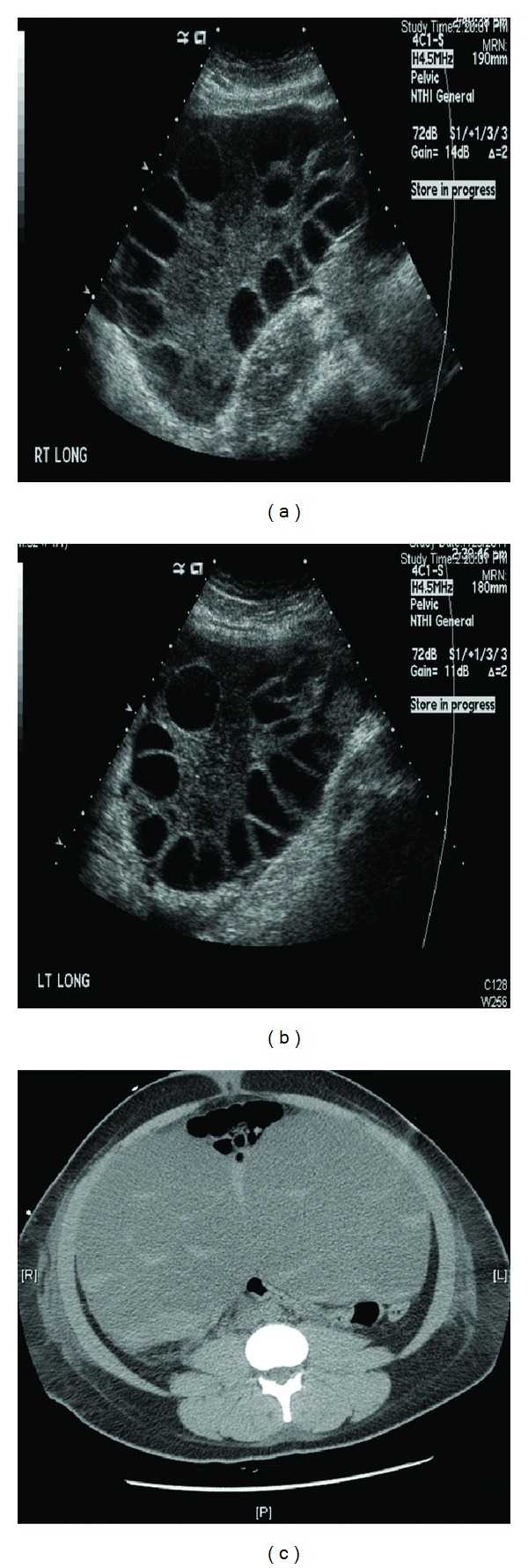
Ultrasound images showing enlarged right and left ovaries with multiple peripherally enlarged follicles. (a) Right ovary measuring 18 × 14 × 11 cm^3^, (b) left ovary measuring 17 × 12 × 12 cm^3^, and (c) computed tomography of the abdomen and pelvis showing bilateral enlarged multilocular and cystic ovaries.

The patient is a 30-year-old obese African-American female, with no significant past medical history who had been married for three years but was nulliparous. She presented with a two-day history of nausea, vomiting, diffuse abdominal pain, abdominal distension, and lower extremity edema. She recently underwent ovulation induction with follicle stimulating hormone and recombinant human chorionic gonadotropin with retrieval of oocytes (8 in total) which was performed just two days prior to her admission. On examination, she was noted to be in moderate distress with a blood pressure of 86/56 mm Hg, heart rate of 110/minute, and respiratory rate of 26/minute with oxygen saturation of 98% on 2 L oxygen. Her abdomen was distended with diffuse tenderness and guarding with evidence of ascites but without peritoneal signs. Her beta-human chorionic gonadotropin (*β*hCG) was negative and her initial estradiol level was 21,000 pg/mL. Her chest roentgenogram showed normal cardiac silhouette, without any infiltrates or effusions. A diagnosis of severe life-threatening OHSS was made and she was started on IV albumin (25 gm/100 mL) and vasopressors (epinephrine 4 mcg/min) in the intensive care unit (ICU). Bedside abdominal ultrasound showed markedly enlarged ovaries (right ovary 18 × 14 × 11 cm^3^, left ovary 17 × 12 × 12 cm^3^) abutting each other at the middle, with multiple peripherally enlarged follicles with good arterial and venous flow signals and moderate amount of ascites and bilateral small pleural effusions (Figures 1(a) and 1(b)). Computed tomography of the abdomen and pelvis showed bilateral enlarged multilocular and cystic ovaries touching each other at the middle with multiple dependent intracystic crescents and truncal edema (Figure 1(c)). Her intra-abdominal pressure (IAP) was elevated at 28 mmHg and she subsequently underwent therapeutic paracentesis under ultrasound guidance with a net removal of 1500 cc of serosanguinous fluid. Her symptoms transiently improved after the paracentesis but the repeat intra-abdominal pressure was still elevated at 24 mm Hg. Her renal function progressively worsened with blood urea nitrogen (BUN) and creatinine levels peaking at 46 mg/dL and 4 mg/dL, respectively, on the 5th ICU day. Her body weight increased from 78 Kg on admission to 98 Kg on the 5th ICU day. Repeat ultrasound of the abdomen and pelvis on the 6th ICU day revealed interval increase in the size of the ovaries (right ovary: 25 × 12 × 25 cm^3^, left ovary: 22 × 11 × 17 cm^3^), with good arterial and venous flow signals, mild ascites, and evidence of hydroureter and hydronephrosis in the left kidney; right kidney appeared to be apparently normal. On ICU day 7, she underwent cystoscopy with retrograde bilateral ureteral stenting and ultrasound-guided aspiration of bilateral ovaries (500 cc of fluid removed). Her symptoms and renal function progressively improved and she was eventually discharged home on hospital day 14.

## 3. Discussion

OHSS is classified as either an early onset OHSS (occurring within 9 days after oocyte retrieval and is related to hyperresponsiveness of the ovaries to gonadotropin stimulation and exogenous hCG) or as a late onset OHSS (appears after this 10-day period and is associated with hCG produced by an implanted embryo) [4, 5]. OHSS can cause a rapid accumulation of volume (from 1.5 to 17 liters) in the peritoneal cavity that can lead to organ dysfunction, including respiratory impairment and oliguria [6]. OHSS causes the ovaries to enlarge and when combined with ascites can overwhelm the compliance of the abdominal wall thereby elevating the intra-abdominal pressure to the point of causing an abdominal compartment syndrome (ACS) [7, 8]. In spite of frequent occurrence of ACS and oliguria in OHSS, acute renal failure secondary to obstructive uropathy is uncommon in OHSS. This patient had severe OHSS that resulted in ascites, ACS, pleural effusions, anasarca, and hypotension. All of these factors could have potentially contributed to oliguria and ARF, but the patient had evidence of obstruction on ultrasound and her symptoms and renal function improved markedly after bilateral ureteral stenting. To date there have been just two case reports of obstructive uropathy associated with OHSS [9, 10]. We therefore suggest that obstructive uropathy should also be considered as a possible etiology in patients with OHSS who develop oliguria or ARF.

## References

[1] Delvinge A, Rozenberg S (2002). Epidemiology and prevention of ovarian hyperstimulation syndrome (OHSS): a review. *Human Reproduction Update*.

[2] Vasseur C, Rodien P, Beau I (2003). A chorionic gonadotropin-sensitive mutation in the follicle-stimulating hormone receptor as a cause of familial gestational spontaneous ovarian hyperstimulation syndrome. *The New England Journal of Medicine*.

[3] Manno M, Tomei F (2008). Renin-angiotensin system activation during severe OHSS: cause or effect?. *Fertility and Sterility*.

[4] Dahl Lyons CA, Wheeler CA, Frishman GN, Hackett RJ, Seifer DB, Haning RV (1994). Early and late presentation of the ovarian hyperstimulation syndrome: two distinct entities with different risk factors. *Human Reproduction*.

[5] Papanikolaou EG, Tournaye H, Verpoest W (2005). Early and late ovarian hyperstimulation syndrome: early pregnancy outcome and profile. *Human Reproduction*.

[6] Grossman LC, Michalakis KG, Browne H, Payson MD, Segars JH (2010). The pathophysiology of ovarian hyperstimulation syndrome: an unrecognized compartment syndrome. *Fertility and Sterility*.

[7] Maslovitz S, Jaffa A, Eytan O (2004). Renal blood flow alteration after paracentesis in women with ovarian hyperstimulation. *Obstetrics and Gynecology*.

[8] Malbrain MLNG, Cheatham ML, Kirkpatrick A (2006). Results from the International Conference of Experts on Intra-abdominal Hypertension and Abdominal Compartment Syndrome. I. Definitions. *Intensive Care Medicine*.

[9] Merrilees DA, Kennedy-Smith A, Robinson RG (2008). Obstructive uropathy as the etiology of renal failure in ovarian hyperstimulation syndrome. *Fertility and Sterility*.

[10] Khalaf Y, Elkington N, Anderson H, Taylor A, Braude P (2000). Ovarian hyperstimulation syndrome and its effect on renal function in a renal transplant patient undergoing IVF treatment: case report. *Human Reproduction*.

